# IgLON 5 Antibody Syndrome: Isolated Case of a Patient With Indolent Disease Progression and Unusual MRI Findings

**DOI:** 10.7759/cureus.13386

**Published:** 2021-02-17

**Authors:** Sihyeong Park, Jonathan Doan, Irfan Sheikh, Ajaz A Sheikh

**Affiliations:** 1 Neurology, University of Toledo Medical Center, Toledo, USA

**Keywords:** iglon-5, leptomeningeal enhancement

## Abstract

IgLON-5 antibody syndrome has a myriad of clinical presentations, including gait instability, movement disorders, abnormal eye movements, bulbar symptoms, sleep disorders, neuropsychiatric symptoms, dysautonomia, and peripheral neuropathy. Abnormal magnetic resonance imaging (MRI) findings have also been reported such as leukoariotic changes and cerebral and cerebellar atrophy. We present a case of IgLON-5 antibody syndrome with a unique MRI finding of persistent leptomeningeal enhancement. A close follow-up would be warranted as the progression of the disease may be indolent.

## Introduction

Although IgLON-5 is functionally a neuronal cell adhesion protein, its exact biochemical role remains to be elucidated. IgLON-5 antibody was initially described in the context of sleep disorders [1]. However, additional characteristic clinical manifestations have been reported to constitute IgLON-5 antibody-associated syndrome. Typical manifestations include gait instability, bulbar symptoms, movement disorders, neuropsychiatric symptoms, diplopia, dysautonomia, and dysarthria [2]. Abnormal magnetic resonance imaging (MRI) findings have also been observed. Common changes have been noted in the brainstem, cerebellum, hypothalamus, and basal ganglia, which are regions known to be affected in tauopathy [3]. Here, we present a case of IgLON-5 antibody disease with a unique MRI finding of persistent leptomeningeal enhancement.

## Case presentation

**Figure 1:**
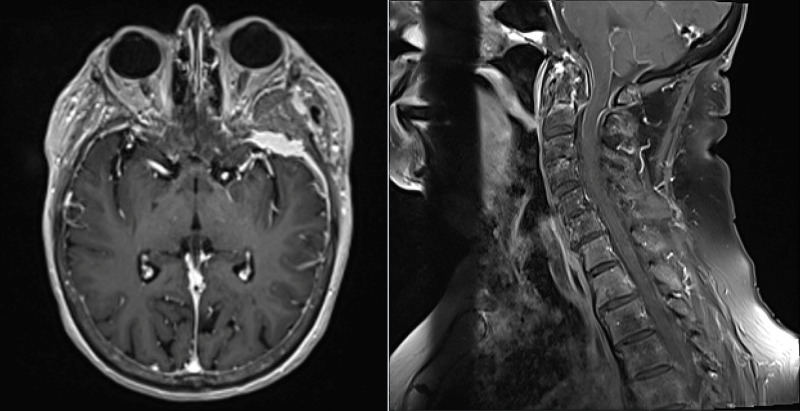
MRI brain and cervical spine with and without contrast. Brain MRI showing a stable appearance of a left temporal meningioma and enhancement along the leptomeninges lining the cerebellum and upper cervical spinal cord. MRI, magnetic resonance imaging

**Figure 2:**
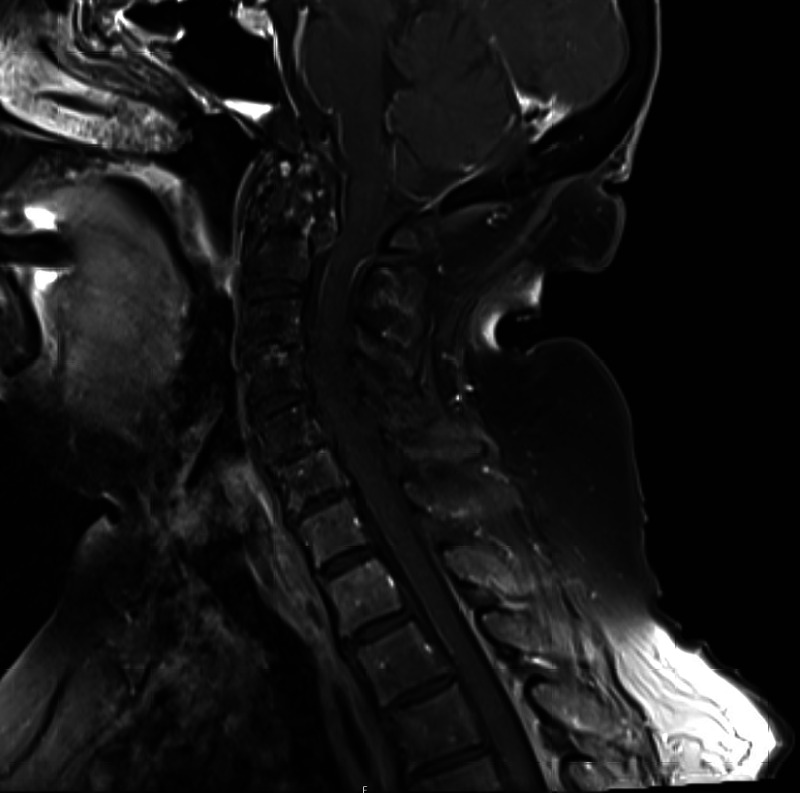
MRI cervical spine with and without contrast nine months later showing persistent meningeal enhancement. MRI, magnetic resonance imaging

An 84-year-old woman, with a history of bladder cancer and left temporal meningioma, presented to the hospital for neck pain related to spondylosis. While initially undergoing evaluation for a C1-2 fusion, brain MRI showed a stable appearance of a left temporal meningioma and enhancement along the leptomeninges lining the cerebellum and upper cervical spinal cord (Figure 1). Subsequently, lumbar puncture was performed, and cerebrospinal fluid (CSF) analysis was unremarkable (white blood cells = 3/µL, red blood cells = 2,235/µL, total protein = 77 mg/dL, and glucose = 70 mg/dL). Small mature-appearing lymphocytes and a few monocytes were noted on cytology, but no malignant cells were identified. Infectious meningitis panel was negative. West Nile virus antibodies (immunoglobulin [Ig]M and IgG) and Lyme IgG were not detected. During tissue immunofluorescence assay screening as part of the paraneoplastic syndrome evaluation, IgLON5-IgG was incidentally detected in the CSF. The finding was confirmed by cell-based assay. Six months later, she followed-up in the clinic and had newly developed mild tremors, xerostomia, difficulty swallowing, and worsening of snoring. Neurological examination was notable for an intention tremor with frequency of 6-8 Hz, which worsened with action. She was diagnosed with anti-IgLON5 antibody disease. Given her dysphagia and snoring, swallow study and polysomnography were recommended. Speech therapy evaluated the patient five months after discharge, and a swallow study was not performed due to difficulty swallowing only with saliva and not food or liquids. Additionally, a previous ultrasound prior to her first hospitalization revealed a complex partially cystic nodule in the right lobe of the thyroid, which may have contributed to her dysphagia. Video fluoroscopy swallow study was recommended to determine if nodules were responsible for the patient’s dysphagia. Additionally, the patient refused undergoing polysomnography at the one-month neurology follow-up. Treatment with steroids was discussed, but the patient declined, and the risks seemed to outweigh the benefits at that time, as symptoms were only mild. A close follow-up was recommended for any change in symptoms. Repeat MRI imaging at nine months showed persistent meningeal enhancement (Figure 2).

At the 12-month follow-up, the patient presented with new complaints of screaming and yelling during sleep, intermittent diplopia, worsening gait with shuffling characteristics, pseudo-bulbar affect, new-onset depression, and memory impairment. Symptoms were concerning for possible progression of IgLON5 antibody syndrome; however, several confounding comorbid conditions, including sleep apnea and sleep disturbance, stalled the overall treatment decision, and close observation was continued. Neurologic examination revealed unchanged hyperkinetic intention tremor and no new neurologic findings. She was advised to follow with neuropsychology to rule out cognitive disease including Alzheimer’s disease. Sertraline had recently been prescribed and recommendations were made to continue it. Again, polysomnography was recommended but was refused by the patient. Steroid treatment was again reconsidered, but was refused by the patient given significant risks involved with worsening arthritis, depression, and bleeding risk secondary to warfarin treatment.

Shortly thereafter, she was admitted to hospital for congestive heart failure exacerbation and a urinary tract infection (UTI) and started experiencing intermittent episodes of somnolence along with incoherent speech and confusion. The fatigue improved upon discharge, but she still had persistent symptoms according to the family. Altered mental status was thought to be secondary to metabolic encephalopathy as a result of her UTI and pain medication (hydrocodone/acetaminophen). However, MRI brain was ordered to rule out other neurologic etiology to explain the patient’s altered mental status. Repeat imaging at 16 months showed persistent leptomeningeal enhancement with no interval changes.

One month after discharge, she reported to be suffering from frequent falls, some of which had caused her to subluxate her right shoulder. Her mental status worsened, and she started to experience neuropsychiatric symptoms. Symptoms included unprovoked crying spells, increased somnolence, anhedonia, dependent personality with an over-reliance on her husband to perform her activities of daily lives, agoraphobia, irritability, and auditory and visual hallucinations. She was again recommended to undergo neuropsychological evaluation; however, she refused due to severe paranoia. Follow-up in the clinic revealed worsening sleep patterns and behaviors including staying up all night and sleeping during the day. She was also experiencing bizarre behaviors, for example, making cappuccino in the middle of the night in conjunction with her previously described neuropsychiatric symptoms. The patient again refused testing and empiric treatment with steroids and immunosuppressive therapy.

## Discussion

IgLON5 is an adhesion molecule widely expressed in the central nervous system (CNS), whose function is poorly understood. Patients presenting with autoimmunity to IgLON5 can present with progressive CNS disorders involving sleep and movement abnormalities. Symptoms of IgLON5 antibody syndrome include gait instability, movement disorders (Parkinsonism, myoclonus, tremor, ataxia, chorea), abnormal eye movements (nystagmus, hypometric saccades), bulbar symptoms (dysphagia, aspiration, orthostatic hypotension), sleep disorders (REM sleep behavior disorder, nocturnal frontal lobe epilepsy, parasomnia), neuropsychiatric symptoms (depression, memory impairment, hallucinations, anxiety), dysautonomia (urinary and bowel incontinence, anhidrosis), and peripheral neuropathy [2]. IgLON5 antibody syndrome can be an insidious process with symptoms taking years to manifest [3]. Our patient presented initially presented with neck pain with an abnormal MRI consistent with leptomeningeal enhancement, a finding not yet reported in IgLON5 antibody syndrome. Her symptoms progressed over the years, with severe neuropsychiatric symptoms and paranoia. Her symptoms further included sleep disturbance suggesting possible REM sleep behavior disorder. Nocturnal frontal lobe epilepsy could not be completely ruled out, given her nocturnal awakenings with crying spells and screaming. She continued to develop worsening gait instability, requiring continued utilization of her walker, depression, and dysphagia.

​MRI of patients with IgLON5 antibody syndrome can include leukoariotic changes and cerebral and cerebellar atrophy [2]. Our patient had unusual findings of persistent leptomeningeal enhancement on MRI that have not been reported in the literature thus far [3,4]. Although there is still a remote possibility that the imaging changes may be secondary to an alternate disease process, such as IgG4-related disease (not tested in our patient), our patient had progressive symptoms that would be otherwise consistent with IgLON5 antibody syndrome. A case of anti-IgLON5 with predominant cerebellar tau deposits in the presence of leptomeningeal inflammation was described, utilizing tau and translocator protein-positron emission tomography imaging [4]. That case, along with the case presented here, can potentially elucidate the pathophysiology behind the leptomeningeal enhancement in patients with IgLON5 antibody syndrome.

​Treatment of this disease so far has no reported algorithmic approach as the evidence is lacking. However, there has been some evidence behind the utilization of immunosuppressive therapy, including corticosteroids, IVIg, and plasma exchange [2]. Our patient was offered immunosuppressive therapy several times, which she refused, given the risks involved and potential for worsening of other co-morbid conditions that would have compromised her overall health. She had primary osteoarthritis of bilateral knees, glenohumeral and hip joints, and atrial fibrillation, and was taking warfarin. Corticosteroids would have increased her risk for fractures, given her recurrent falls, as well as increased the risk of gastrointestinal bleeding, given the concomitant use of warfarin.

​Prognosis of patients with confirmed IgLON5 antibody syndrome remains poor secondary to neuropathologic findings consistent with a tauopathy, with progression to death as a result of respiratory failure.

​This case gives premise to the significant multisystemic abnormalities caused by IgLON5 antibody syndrome. We also describe persistent leptomeningeal enhancement in the context of IgLON5 antibody syndrome, which has not been described before.

Furthermore, IgLON5 antibody syndrome is a rare disease and is often investigated if there is clinical suspicion of an autoimmune pathology, especially in the setting of an underlying malignancy. Our patient had an isolated positive test in the setting of minimal clinical signs and symptoms suggestive of IgLON5 antibody syndrome. However, when prospectively followed on time, clinical signs and symptoms of IgLON5 antibody syndrome became more apparent. The authors conclude that given an isolated positive test in the setting of minimal or no clinical signs or symptoms of IgLON5 antibody syndrome, prospective follow-up should be undertaken to monitor for disease severity and progression. Studies have shown benefit with immunosuppressive therapy acutely with corticosteroids, IVIg, and plasma exchange [4]. Immune modulators could also be considered such as azathioprine, rituximab, and mycophenolate mofetil [4]. If clinically indicated, immunosuppressive therapy should be discussed with the patient prior to administration to avoid any complications. Our patient had significant co-morbidities for which immunosuppressive therapy outweighed the benefits with the potential risk for complications leading to significant morbidity and thus were avoided. Further prospective studies are warranted when it comes to appropriate follow-up and management of patients with IgLON5 antibody syndrome.

## Conclusions

We describe a patient with IgLON-5 antibody syndrome whose MRI demonstrated a novel finding of persistent leptomeningeal enhancement. As we have observed an indolent progression of the disease with an isolated positive test upon presentation, a close follow-up would be warranted.
